# A new machine learning approach for predicting likelihood of recurrence following ablation for atrial fibrillation from CT

**DOI:** 10.1186/s12880-021-00578-4

**Published:** 2021-03-09

**Authors:** Thomas Atta-Fosu, Michael LaBarbera, Soumya Ghose, Paul Schoenhagen, Walid Saliba, Patrick J. Tchou, Bruce D. Lindsay, Milind Y. Desai, Deborah Kwon, Mina K. Chung, Anant Madabhushi

**Affiliations:** 1 Center for Computational Imaging and Personalized Diagnostics, Department of Biomedical Engineering, Case Western Reserve University, 2071 Martin Luther King Drive, Cleveland, OH 44106-7207 USA; 2 Cleveland Clinic Lerner College of Medicine of Case Western Reserve University, Cleveland, OH USA; 3 Department of Cardiovascular Medicine, Heart, Vascular and Thoracic Institute, Cleveland Clinic, Cleveland, OH USA; 4 Department of Cardiovascular and Metabolic Sciences, Lerner Research Institute, Cleveland Clinic, Cleveland, OH USA; 5 Louis Stokes Cleveland Veterans Administration Medical Center, Cleveland, OH USA

**Keywords:** Atrial fibrillation, Left atrium, Atrial fibrillation recurrence, Atrial shape morphology, Atrial radiomic features, Shape differentiation

## Abstract

**Objective:**

To investigate left atrial shape differences on CT scans of atrial fibrillation (AF) patients with (AF+) versus without (AF−) post-ablation recurrence and whether these shape differences predict AF recurrence.

**Methods:**

This retrospective study included 68 AF patients who had pre-catheter ablation cardiac CT scans with contrast. AF recurrence was defined at 1 year, excluding a 3-month post-ablation blanking period. After creating atlases of atrial models from segmented AF+ and AF− CT images, an atlas-based implicit shape differentiation method was used to identify surface of interest (SOI). After registering the SOI to each patient model, statistics of the deformation on the SOI were used to create shape descriptors. The performance in predicting AF recurrence using shape features at and outside the SOI and eight clinical factors (age, sex, left atrial volume, left ventricular ejection fraction, body mass index, sinus rhythm, and AF type [persistent vs paroxysmal], catheter-ablation type [Cryoablation vs Irrigated RF]) were compared using 100 runs of fivefold cross validation.

**Results:**

Differences in atrial shape were found surrounding the pulmonary vein ostia and the base of the left atrial appendage. In the prediction of AF recurrence, the area under the receiver-operating characteristics curve (AUC) was 0.67 for shape features from the SOI, 0.58 for shape features outside the SOI, 0.71 for the clinical parameters, and 0.78 combining shape and clinical features.

**Conclusion:**

Differences in left atrial shape were identified between AF recurrent and non-recurrent patients using pre-procedure CT scans. New radiomic features corresponding to the differences in shape were found to predict post-ablation AF recurrence.

## Background

Atrial fibrillation (AF) is a common cardiac arrhythmia in which rapid and irregular electrical atrial activation causes loss of synchronized contraction of the atria. Potential consequences include symptoms from the rapid and irregular conduction to the ventricle, loss of atrioventricular synchrony, and risk of thromboembolic complications, such as stroke. Rhythm control of AF typically centers on suppression with antiarrhythmic drugs or catheter ablation, the latter primarily directed toward isolation of the pulmonary vein ostia, where initiating triggers have been observed. For persistent or long-standing persistent AF, the success of ablation can be limited: up to 80% may recur within a year [1], and for this population there is controversy over whether additional substrate ablation should be performed beyond pulmonary vein (PV) isolation. Consequently, there is interest in predicting the likelihood of recurrence from pre-ablation contrast-enhanced computed tomography angiogram (CE-CTA) scans, which may aid in patient selection for ablation and in procedure and post-procedure planning. Interrogation of quantifiable effects of structural parameters through radiologic biomarkers from pre-ablation CE-CTA scans may improve patient and procedural stratification.

Previous studies have identified left atrial measurements such as volume [2–4], anterior–posterior (AP) diameter [5], and atrial sphericity [6] as predictors of AF recurrence. However, there is a lack of consensus on the strength of these individual biomarkers in gauging the risk of AF recurrence. These studies typically have not considered the comparative role of the different sites of the atrium in AF recurrence. For instance, it is postulated that AF-induced atrial remodeling is asymmetric due to the abnormal non-homogeneous mechanisms of AF [2], and consequently, different sites of the left atrium will most likely have differential prognostic characteristics. This motivates the need for studying differential prognostic characteristics of the left atrium (LA) to predict likelihood of post-ablation recurrence from pre-operative scans.

We tested the hypotheses that factors that contribute to AF recurrence after ablation induce differential left atrial remodeling which can be interrogated using pre-ablation scans, and that the significant sites of differentiation can be transformed into quantitative features which are associated with ablation outcome. In this paper we present a new statistical and machine learning approach, the Differential Atlas for Identifying Sites Predictive of Recurrence, (DiSRn) to address the two questions of (1) whether and where there exist differences between LA shape of post-ablation AF recurrent (AF+) and non-recurrence (AF−) patients, and (2) how these shape differences may be transformed into quantitative descriptors for predicting recurrence using pre-ablation CE-CTA scans.

## Methods

### Patient selection

This was a retrospective observational study of patients who underwent catheter-based pulmonary vein isolation for AF at the Cleveland Clinic between July 2015 and November 2016. Inclusion criteria included history of AF and pulmonary vein CE-CTA obtained prior to AF catheter ablation procedures performed by staff who routinely obtained pre-procedure CE-CTA scans during the inclusion time period. Both cryoballoon and radiofrequency ablation procedures were included. The study was approved by the Cleveland Clinic Institutional Review Board for retrospective medical records and imaging review and performed in accordance with institutional guidelines.

**Fig. 1:**
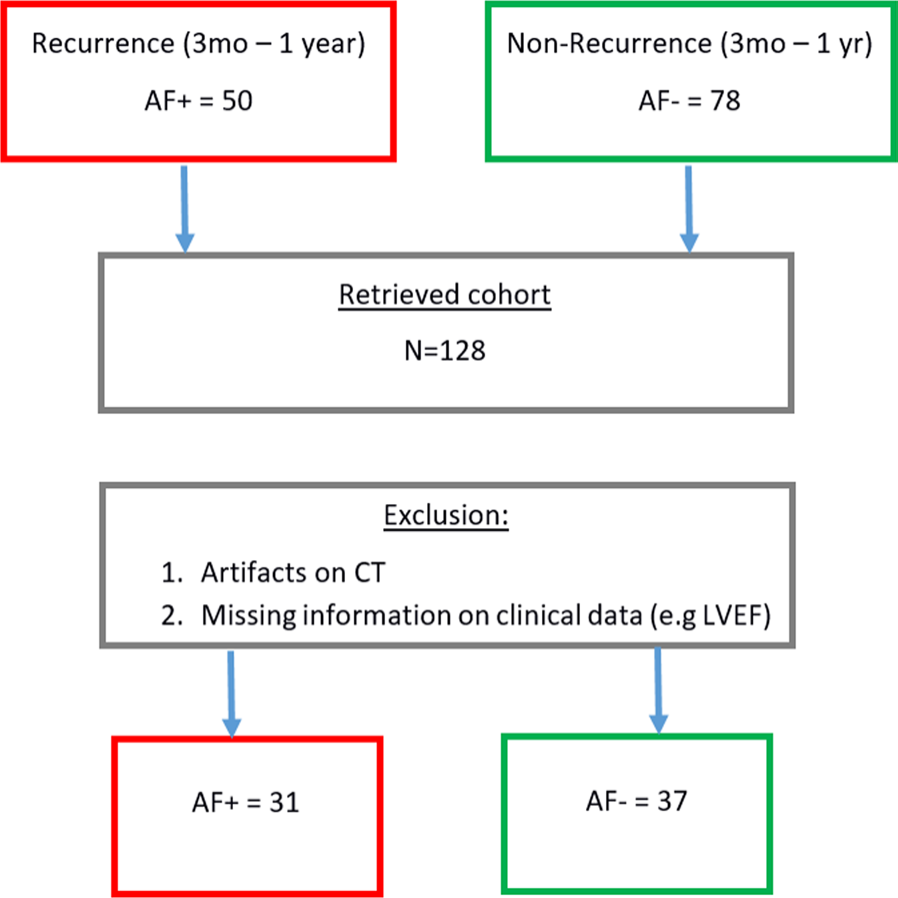
Patient selection for analysis. The Initial representation of (48%) recurrent patients in the sample is an effect of selection bias aimed at minimizing class imbalance

Subjects were excluded who had prior ablation, congenital heart disease, and valvular disease. Out of 128 patients remaining, we further excluded scans with significant artifact, and those with limited contrast making left atrium boundaries blurry (and consequently inaccurate segmented LA boundaries—essential for Shape analysis), leaving a total of 68 patients. This final set consisted of 37 who had no recurrence versus 31 who had recurrence between 3 months to 1 year post-ablation, as determined by clinical assessment usually documented by ECGs or ambulatory monitoring. The 3-month blanking period is a standard in determining recurrence rates after AF [7]. The patient selection process is summarized in Fig. 1.

### Multidetector CT acquisition

Multidetector CT was performed using multiple scanner technologies with similar protocols (Definition Flash or Sensation 64, Siemens Medical Solutions, Erlangen, Germany, or iCT, Philips Medical Systems, Best, the Netherlands). Low-osmolar nonionic contrast agent (70–90 mL at flow rates between 3.5 and 4.5 mL/s) was injected into the antecubital vein using an 18-gauge needle and a power injector. Bolus tracking technique was used to appropriately time the onset of image acquisition. After contrast injection, a prospectively ECG-triggered scan in a systolic phase (specifically targeted to late systole, where the atrial volume is expected to be close to maximum) was performed covering the region immediately beneath the aortic arch to the apex of the left ventricle. Rate control was not performed.

### General approach

Our approach consisted of two main steps: (1) identification of Surface of Interest (SOI), and (2) diffeomorphometric Feature map extraction from the SOI. The SOI identification was based on a statistical shape differentiation method which compares and differentiates atlases from two groups, and has been successful in the study of post-therapy biochemical recurrence of prostate cancer patients [8], as well as the study of prostate shape differences between normal and malignant cancer on MRI [9]. This step consisted of an initial atlas creation from pre-segmented LA masks of AF+ and AF− patient CE-CTA scans, followed by a Generalized Linear Model (GLM) based t-testing for the SOI.

The feature extraction step also consisted of computing an optimal deformation field between template SOI and patient SOI, followed by computation of the curl and magnitude of the deformation field on the SOI.

The constructed feature set was used to evaluate post-ablation outcome using a cross-validation model built from gradient boosted classifier (xgboost) [10]. This classifier is a classification and regression tree based algorithm that creates a meta-classifier from several weak (short depth) learners using an aggregation score.

### Identification of surface of interest

#### Left atrium segmentation and atlas creation

The construction of the LA model from CE-CTA scan for each patient was achieved by an interactive semi-automated segmentation method developed in-house. The segmentation algorithm is based on the curvature-based seeded watershed segmentation method introduced in [11]. The toolbox also enables additional steps of clipping of the pulmonary veins and the left atrial appendage to obtain a final segmentation of the full left atrial volume for ease of subsequent shape differential estimation. The clipping of the pulmonary veins was done 1–2 cm from the ostia. For the atrial appendage the toolbox enables selection of three planar points around the ostium, which are used to form a slicing plane to separate the appendage from the left atrium.

Fifteen samples were randomly selected from the AF+ and AF− groups respectively. We constructed template LA for the cohort, AF+ and AF− using respective median volume LA (T, T+ and T− respectively). All patients with recurrence were registered to the T+ template to create an AF+ atlas. Similarly, all patients without recurrence were registered to the T− template to create an AF− atlas. A block matching strategy [12] was adopted to determine the transformation parameters for the affine registration. The affine registration of the moving image to the reference image was followed by a B-spline [13] based non-rigid registration scheme. Segmented LA masks were used to constrain the registration to within the volume of interest. The segmented masks of the LA were given the same transformation as the registered images to bring the LA masks and surfaces into correspondence.

#### Experiment 1: sites of interest (SOI) estimation

To perform a statistical comparison of the LA shape between AF+ and AF− atlases, the registered LA masks of both groups were isotropically scaled to 1 mm resolution. An implicit representation of the LA surface was obtained using the signed distance representation, which enabled a t-test based comparison of the shape via a non-parametric General Linear Model (GLM) based t-test framework [14]. Statistically significant shape differences of the LA surfaces were quantified with 5000 random permutation testing with p-values corrected for multiple comparisons. Significant shape differences between AF+ and AF− cohorts were then identified as constituting the SOI, and mapped to cohort template T. Detailed description of this method may be found in [8].

#### Diffeomorphometric feature map extraction

**Fig. 2:**
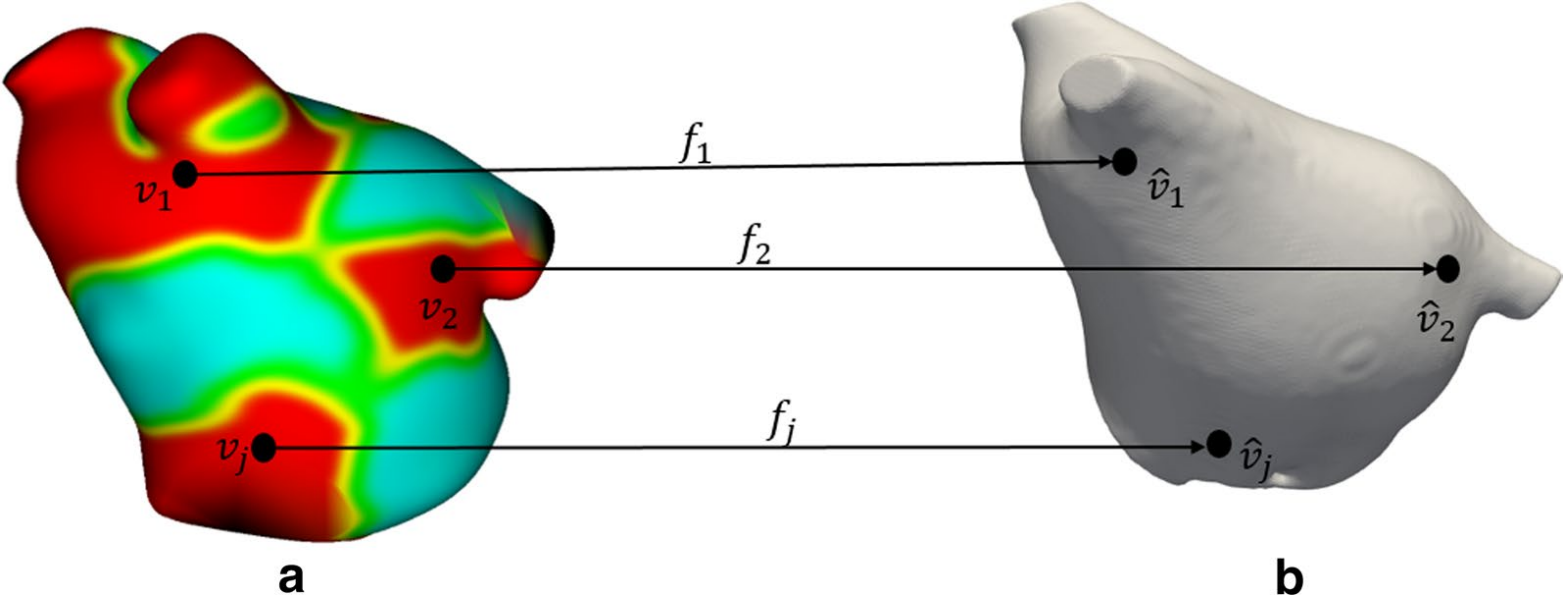
The left image shows the template atlas with indicated SOI (red regions) where there is statistically significant (*p* < 0.05) difference between atrial shape of AF+ and AF−. The set belonging to the SOI is mapped to corresponding set on a sample patient’s atrium (right image) during registration

We developed a new class of features based on statistical descriptors of the deformation field obtained after registering the template LA (T) to the patients’ LA models. The order of registration (from representative model to the respective patient model) allows us to track and map the trajectories of the SOI to patient models as illustrated in Fig. 2.

Once the template was registered to the respective patient models, we extracted the optimal displacement vectors to create a feature map based on the deformation field. The feature map comprised the magnitude and curl of the deformation field. In particular, the norm of the deformation field is equivalent to the amount of energy that is expended to distort template SOI to the patient SOI, while the curl represents the rotational acceleration that should be realized to transform the template SOI to patients’ SOI.

**Fig. 3:**
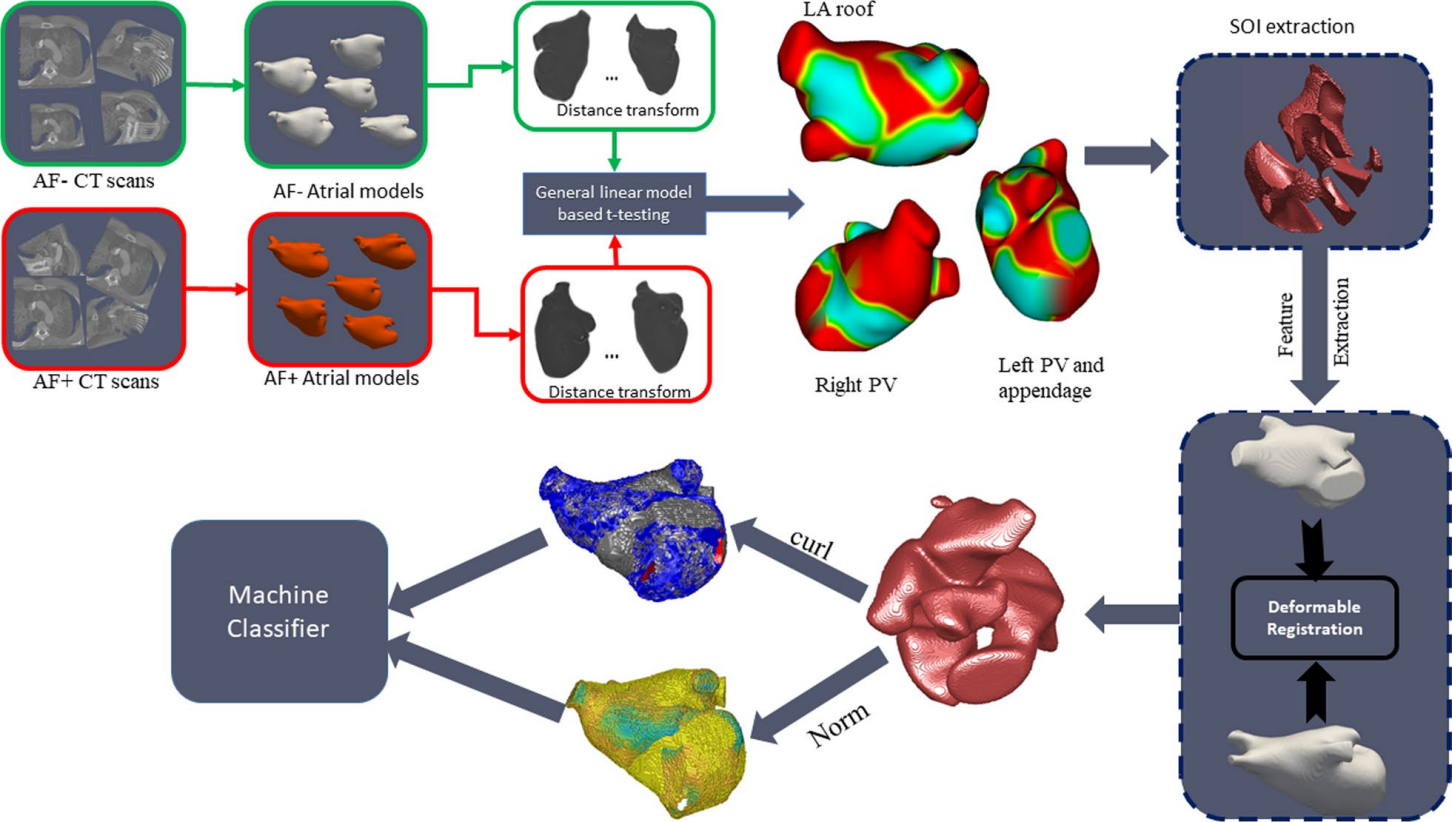
Pipeline for atrial shape differentiation for AF recurrence

The magnitude of deformation was captured in terms of four (4) first order statistics including the minimum, mean, maximum and standard deviation of the norm of x, y, z displacements across the mesh points on the SOI. We also include the mean displacements of each component coordinate x, y, and z. For the curl features, the minimum, mean and maximum statistics of each component (x, y, z) of the curl across the SOI mesh were calculated (these features can then be used for predicting ablation outcome). The process described above is illustrated in Fig. 3.

In summary, the purpose of Experiment 1 is twofold: (1) compute the template SOI, to show the areas of the atrial surface that are significantly different in shape between AF+ and AF−, and (2) map the template SOI to each patient LA, and extract deformation features from their respective patient SOI. These features will then be used to demonstrate an association between the detected SOI (areas on the LA, around PVs) and ablation outcome.

#### Experiment 2: diffeormorphormetric features on SOI versus outside the SOI

The purpose of this experiment is to show that the estimated SOI in Experiment 2 is closely associated with ablation outcome than the remaining parts of the atrial surface (cSOI). For this purpose, the same class of features in Experiment 2 was extracted from the areas outside the SOI (cSOI). A gradient boosted classifier was trained and evaluated in 100 runs of stratified fivefold cross validation on the remaining 67 patient set with the template (T) removed. We removed the template because each patient feature as computed in Experiment 1 consist of displacement properties between the template T and the patient Las. However no meaningful displacement between the template and itself was identified. Hence the scan employed for the template was not used as part of the cross-validation.

#### Experiment 3: SOI features and clinical parameters

The performance of clinical parameters associated with AF recurrence were compared against the SOI features. These parameters included age, sex, left atrial volume (LAV), left ventricular ejection fraction (LVEF), body mass index (BMI), sinus rhythm at the time of ablation, AF type (paroxysmal vs. persistent), and catheter-ablation type (Cryo vs RF), clinical factors that have been reported to be predictive of AF ablation success [7, 15]. We used the clinical features to build a gradient boosted classifier (${XGB}^{cl}$), while the shape features were used to build another gradient boosted classifier (${XGB}^{s}$). The two feature sets were also combined to build a third classifier, ${XGB}^{s+cl}$.

## Results

### Patient population

The cohort used in the study consisted of 68 patients, with 37 having recurrence of AF within the first year of ablation and 31 not having recurrence. Clinical characteristics by AF ablation outcome are shown in Table 1. Univariate analyses showed that patients who had AF recurrence had higher LVEF and older age with a trend toward less sinus rhythm at the time of ablation.

**Table 1:** Comparison of clinical characteristics by outcome for dataset

Characteristic	No recurrence at 1 Year	Recurrence at 1 Year	*p* value
No. of patients	37	31	–
LVEF (%)	54.5 ± 8.65	58.4 ± 5.94	**0.031**
BMI (kg/m^2^)	28.9 ± 5.2	31.4 ± 5.82	0.066
Age (years)	62.6 ± 9.46	68.0 ± 8.24	**0.015**
LA volume (cm^3^)	153.5 ± 42.44	157.3 ± 45.14	0.72
Male	28 (76%)	19 (61%)	0.29
Baseline sinus rhythm	28 (76%)	17 (55%)	0.08
Persistent AF	9 (24%)	11 (35%)	0.42
Cryoablation	10 (27%)	6 (19%)	0.58

Bold values indicate patient characteristics which are statistically significant (*p*-value < 0.05)

### Experiment 1: atrial shape difference

We used a balanced set of 15 random samples each from the AF+ and AF− groups, and the template atrial atlas was taken as the AF− patient atrium with median volume.

**Fig. 4:**
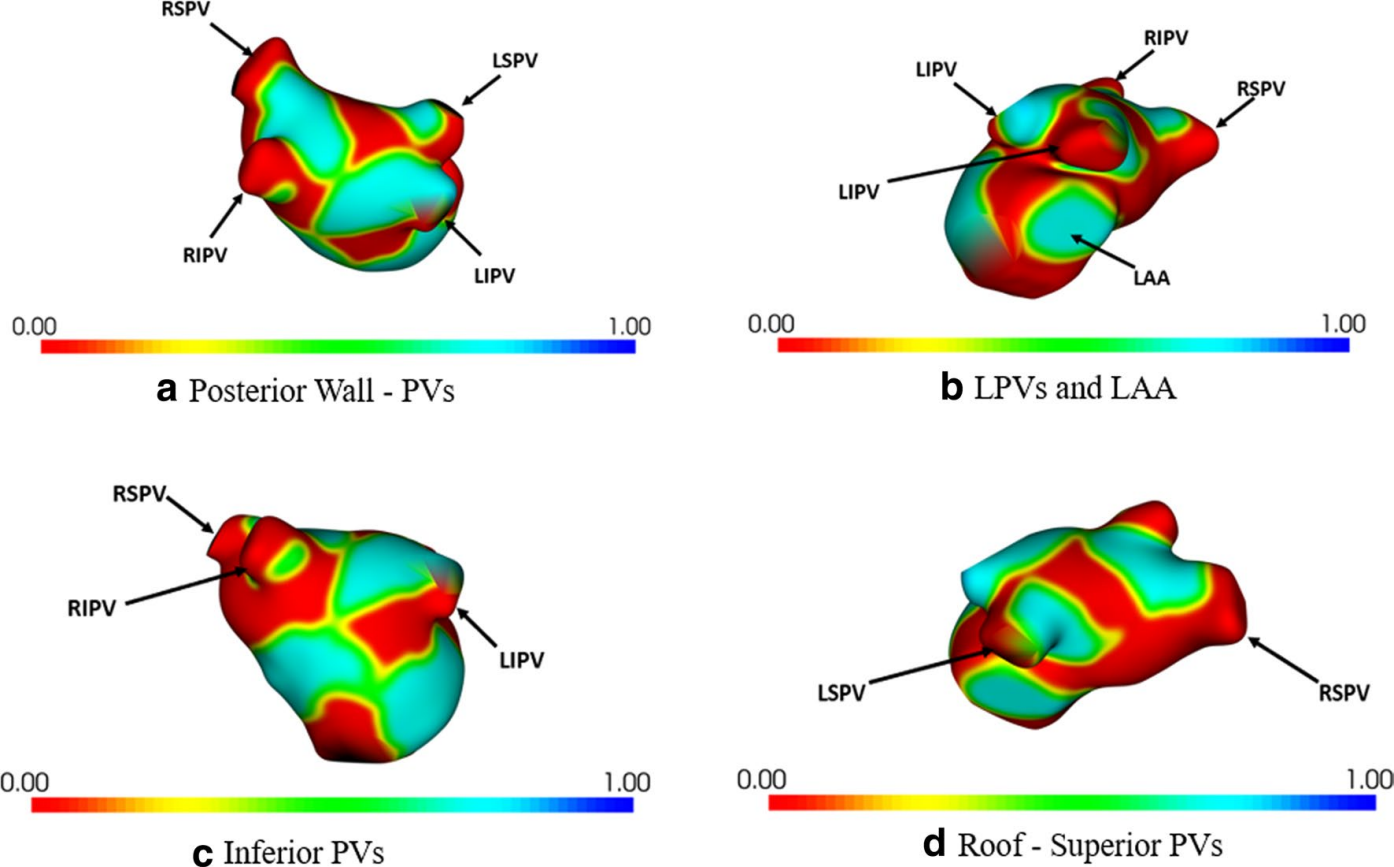
Statistically significant sites of the atrial surface that differ between patients with recurrence and those without. This includes the sites around and between the pulmonary vein ostia, and the region around the base of the left atrial appendage. RSPV—Right superior pulmonary vein; LSPV—left superior pulmonary vein; RIPV—right inferior pulmonary vein; LIPV—left inferior pulmonary vein; LAA—left atrial appendage

Using the implicit shape representation of the balanced set, sites on the atrial surface that had statistically significant (*p* < 0.01) local morphological difference between the two groups were identified as the SOI. These sites included the regions around and between the pulmonary vein ostia of both left and right PVs (often the target sites of ablation). Other significant sites included the regions around the base of the left atrial appendage. These statistically significant sites were projected onto the representative atrial atlas, and then visualized as red-colored surface patches in Fig. 4.

### Experiment 2: comparing DiSRn features within and outside the SOI

A gradient boosted classifier was used for training and testing 100 runs of stratified fivefold cross validation. For each run of the cross validation, the predicted probabilities of recurrence were computed per patient, which were used to compute the average prediction probability for each patient across the runs. The average probabilities were then used to plot the ROC curves and corresponding AUC metrics.

**Fig. 5:**
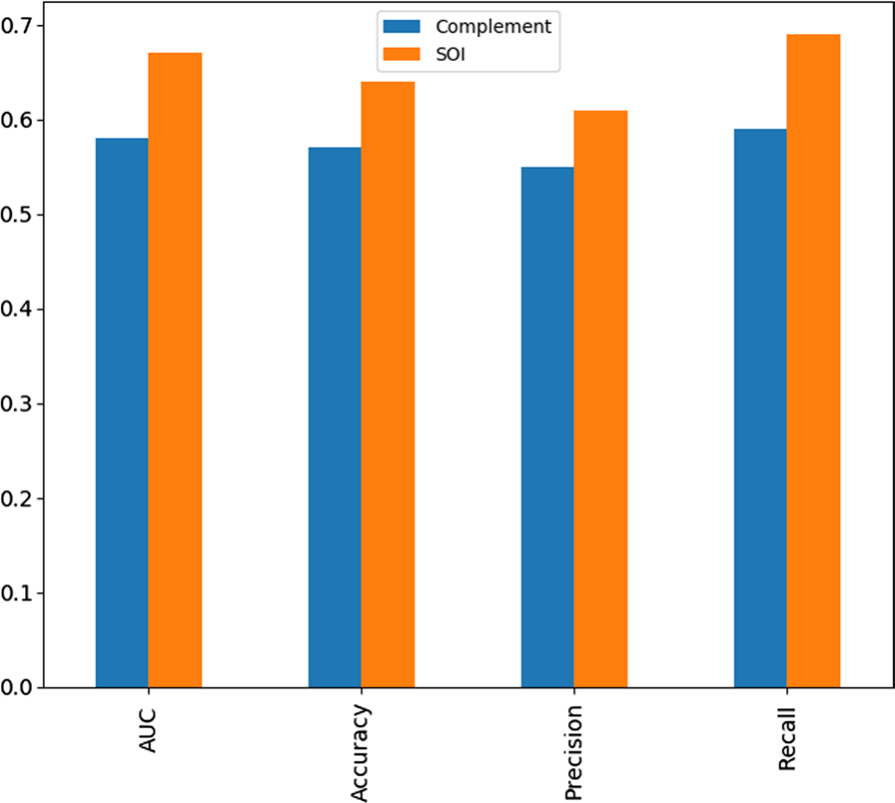
Comparison of SOI features vs Compliment Site features in four performance metrics. The SOI features performs better in all four metrics

Using the cross-validation approach above, we compared the performance of the classifier trained on the shape features from the SOI (${XGB}^{s}$) versus one trained on features outside the SOI (${XGB}^{cs}$), to see the relative prognostic significance of the SOI compared to the remaining sites of the atrial surface. We recorded higher AUC (0.67), accuracy (0.64), precision (0.61) and recall (0.69) for ${XGB}^{s}$ versus lower AUC (0.58), accuracy (0.57), precision (0.55) and recall (0.59) for ${XGB}^{cs}$. This comparison is shown in the bar charts of Fig. 5.

### Experiment 3: evaluating the performance of DiSRn features with clinical factors

The shape-based features extracted with reference to the identified SOI were evaluated against clinical parameters using the AUC of gradient boosted classifiers trained on the shape and clinical factors.

**Fig. 6:**
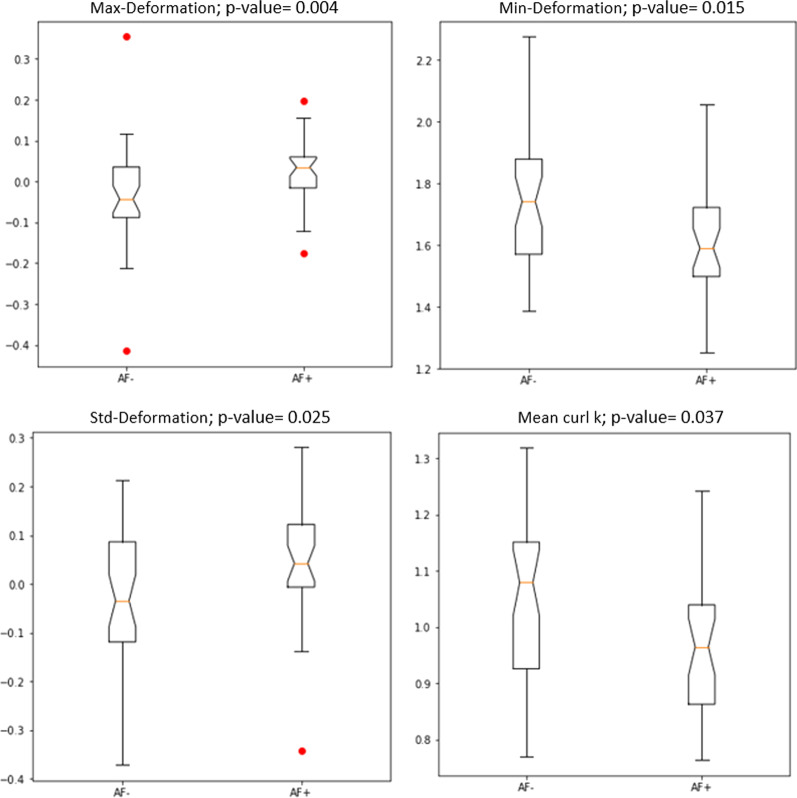
Boxplot of shape features that are significant using the Wilcoxon Rank-sum test

Of the 16 shape features evaluated, Wilcoxon rank-sum testing was used to identify those that were statistically significant (*p* < 0.05). Four features (Max deformation, Min deformation, Std Deormation, and Mean curl component k) were found to be significant and are shown in Fig. 6.

**Fig. 7:**
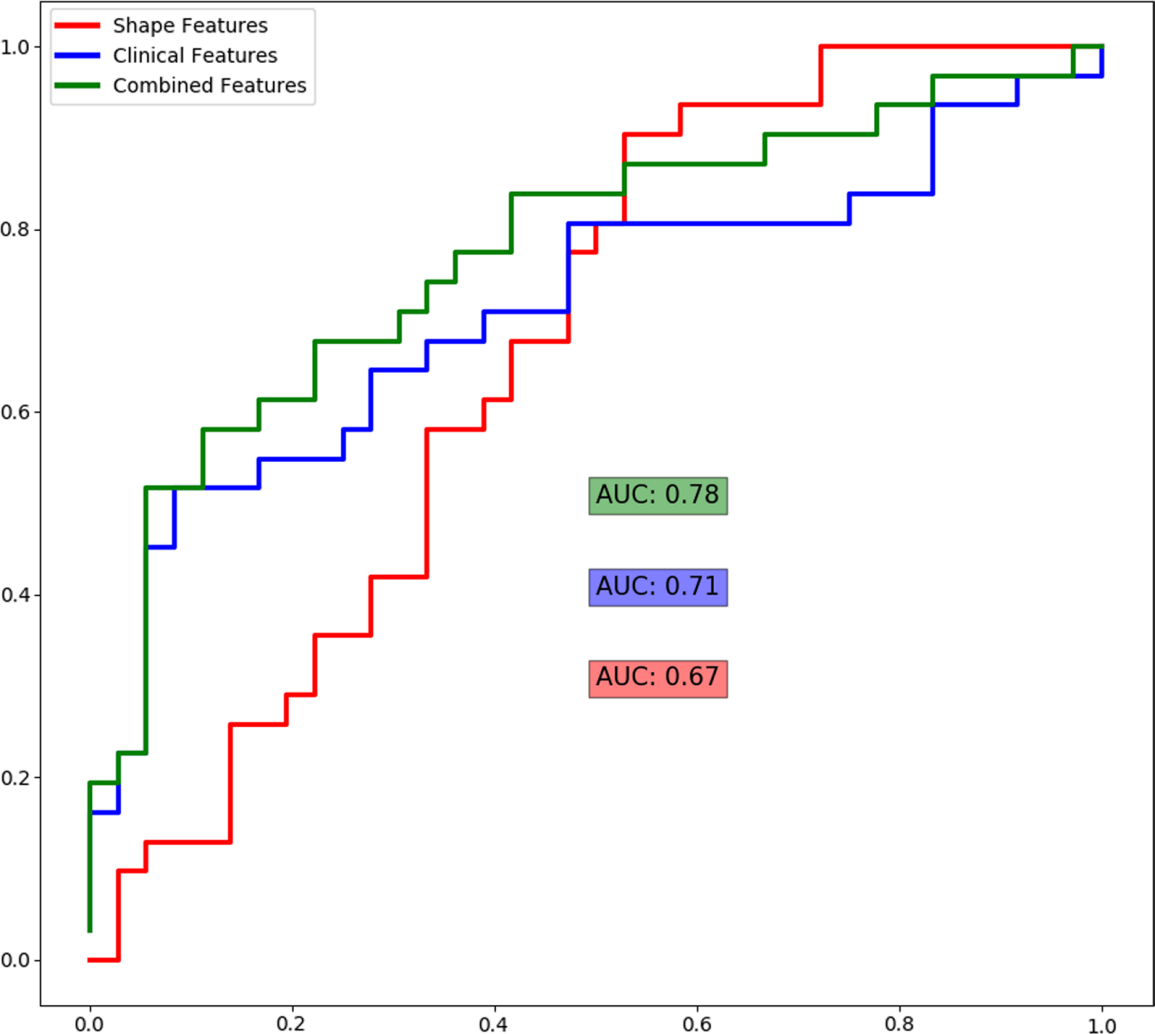
Average ROC curve and AUC over 100 runs of stratified fivefold cross validation. The cross-validation scores are generated using the top four features from shape category, and all clinical features

Using the 4 statistically significant shape features in the cross-validation runs produced an AUC of 0.67. Using the clinical variables in the same cross-validation runs also produced an AUC of 0.71. The composite of top shape features and clinical parameters produced an AUC of 0.78. The corresponding ROC and AUCs are shown in Fig. 7. A test for association between ablation technique (cryoballoon ablation vs. radiofrequency ablation) and outcome was not statistically significant (*p* = 0.58) for this cohort.

## Discussion

Recent studies of AF recurrence have been motivated by the atrial remodeling effects of AF resulting in imaging biomarkers such as atrial volume and anterior–posterior diameter. Due to heterogeneous atrial remodeling [2, 16–19], there is interest in highlighting and studying the differential effects of local morphology of the atrium, and also to distinguish the roles of the different sites and their prognostic significance in post-ablation recurrence. There is established experimental evidence [17–20] of structural atrial remodeling at the myocyte level, largely seen as increase in cell size, perinuclear accumulation of glycogen, and myolysis. However, this process is heterogeneously distributed, with varying effects seen across different cells [17]. This reasonably underpins probable structural changes in atrial morphology [21] and the nature of these structural changes may have prognostic implications for ablation outcome.

The atrial remodeling effect has inspired development of various computer derived imaging markers, with several variants of shape characterization proposed for predicting post ablation recurrence. The left atrial sphericity—a measure of how closely the atrium resembles a sphere—was introduced by Brisbal et al. [6] to quantify remodeling of atrial morphology by MRI and to predict post-ablation recurrence. Jia et al. [3], mapped diffeomorphic features of patients’ atrium on pre-ablation images relative to a constructed atlas to predict AF recurrence using a partial least square fitting. In a recent study by Bieging et al. [22], a particle based modeling (PBM) approach from cardiac magnetic resonance scans done in the DE-MRI-Guided Ablation vs. Conventional Catheter Ablation of Atrial Fibrillation (DECAAF) study was used to generate shape descriptors of patient LA, consisting of principal components of optimal LA mesh points. Similar to the study presented in this paper, these shape-based biomarkers are intrinsically underpinned by left atrial remodeling, but they do not address the structured and differentiated effects of AF on the atrium that may contribute to recurrence.

Our approach differs from these and several other LA shape based methods for recurrence. We first identify shape differences—local sites on the atrial surface that significantly differ in morphology—between AF+ and AF−, and then evaluate the prognostic significance of these sites by translating into shape based features. The process of identifying the sites of significant difference between AF+ and AF− is based on a robust statistical testing approach to finding differences in implicit shapes atlases [8]. This shape interrogation technique was developed to establish the existence of prostate shape differences between patients who have biochemical recurrence after prostatectomy or radiation therapy, where differences in shape were attributed to mechanical forces exerted by aggressive tumors on the tumor microenvironment. Similar to our findings, micro remodeling of tissue may manifest as reconfiguration or dilation of the reference organs, in our case the left atrium.

Our findings suggest that there are morphological shape differences between AF+ and AF− groups, and that shaped based atrial feature maps on these SOI can independently predict AF recurrence. Moreover, this demonstrates that non-uniform changes in the atrial morphology induced by AF may result in microvariations on the left atrial surface, and that these changes can be characterized by the shape and diffeomorphic features. Identifying the source and type of these shape changes will be a focus of future studies that might then impact ablation strategies.

That regions around the pulmonary veins and base of the left atrial appendage, rather than areas on the LA body, were identified to be different between AF recurrent and AF non-recurrent patients is of great interest. These findings were identified on CE-CTAs obtained prior to ablation and so may not be attributable to ablation effects, nor method of ablation, as both radiofrequency and cryoballoon methods were used. It is of interest that the top genetic loci associated with AF risk by genome-wide association studies are on chromosome 4q25 near a gene, *PITX2*, [23, 24] implicated in formation of the pulmonary veins during development [25]. AF risk variants at these loci have been associated with AF recurrence after ablation in many, though not all studies [26–33]. To date the functional connection of this region to AF susceptibility remains elusive. However, the pulmonary vein—left atrial junctions identified by our study support the intriguing concept that these genetic variants might contribute to morphological features in these areas that promote recurrence after ablation. Future planned genomic—radiomic analyses may yield insights or help to identify the features that contribute to the regional morphologic differences.

**Fig. 8:**
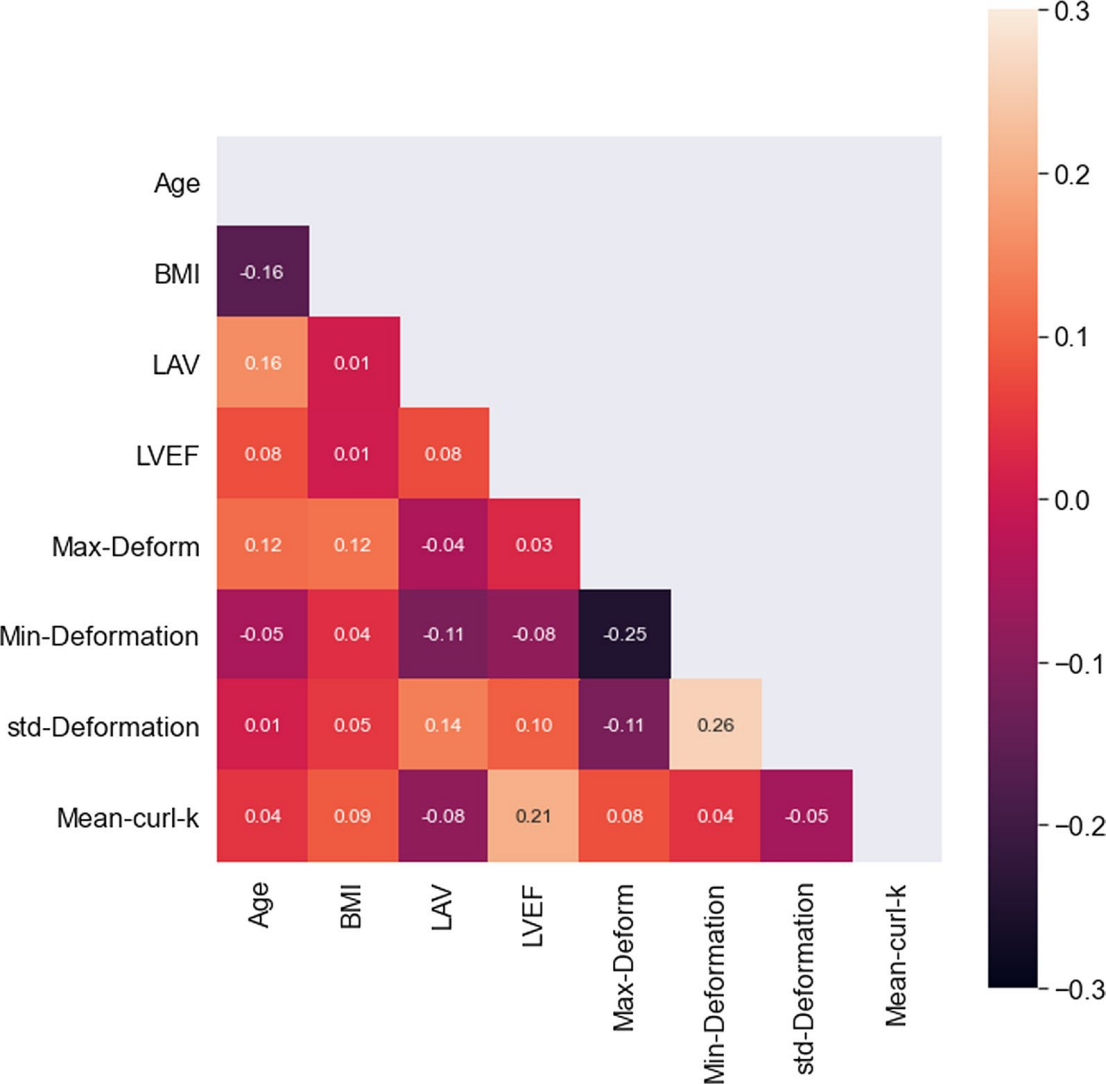
A signed correlation map of the numeric attributes. The top 4 shape features do not show marked correlation with the numeric clinical factors. The heatmap represent the absolute value of the correlation to highlight magnitude of correlation

We note that the clinical parameters considered—age, sex, LAV, LVEF, BMI, sinus rhythm and AF type are linked to ablation outcome [34] and were confirmed here as having good predictive value. The marginal addition of shape-based features to these clinical parameters resulted in modest but appreciable improvement in prediction. Patient age, LAV, LVEF and BMI likely contribute or are related to structural remodeling of the left atrium [35] and may be potential confounders with the shape based features; however we did not see marked correlation between these factors and the shape features, as shown in the correlation heatmap in Fig. 8. These studies show that such analyses of atrial and pulmonary vein shape prior to ablation have the potential to improve prediction of outcome. Additionally, shape features have implications for further studies to determine what and how these features affect AF ablation outcome. Prediction of ablation outcomes using the proposed method needs to be validated in a prospective study.

## Limitations

We acknowledge key limitations of the study. The accuracy of atrial reconstruction is an important step in the estimation of the SOI, and robustness of the segmentation algorithm is crucial. The input CE-CTA images, however, have varying intensity profiles, which causes some variability in the final output LA segmentation. While these variations were less profound in the final cohort used, and our segmentation results were independently verified by a cardiac imaging research assistant, a consistent and perhaps human annotated atrium might minimize concern of effects of variable LA models on future studies. Moreover, while it would be insightful to validate on a larger cohort study, the identified SOI is statistically significant, and it is remarkable that areas of difference could be found with our sample size. The limitation in sample size was due to limited availability of reliably segmented patient atria. There is an inherent dependence of the watershed segmentation algorithm on high contrast images, and this led to the segmentation toolkit producing some unsuitable LA segmentations due to limited contrast and were consequently excluded from subsequent analysis. We hope to address this by adding more samples in the future, as well as adopt a more robust segmentation toolkit. We also recognize the need for controlling for other factors such as potential effects of ablation techniques on recurrence. Controlling for these factors presented a limitation with sample size, and we hope to validate our findings in this study on a larger independent validation dataset. Another experiment to consider in the future is to test the robustness of the SOI detection on a non-AF patient set. This would give further insight into the stability of the proposed SOI detection method. We also note that the baseline rhythm was recorded at the time of ablation, rather than at the time of CT acquisition. It is possible that bias could have been introduced if CTs were excluded from analysis due to poor quality from AF rhythm at the time of acquistion. However, our dataset did not include significant representation from patients in persistent AF (38% in original cohort, and 28% in study experiments). We also acknowledge that due to the limited suitable sample size, the need to maintain class balance for the training of the machine learning algorithm resulted in an over representation of recurrent patients (about 48%). This recurrence rate of 48% is not typical of patients with AFib undergoing ablation. In addition, a test of significance of the combined (shape and clinical) model over the clinical model yielded a p-value of 0.081 using the nonparametric approach proposed in [36]. While this shows the difference between the two AUCs is not significant, the findings suggest that with an increased sample size, we may be able to demonstrate the significantly improved performance of the combined model over the baseline clinical model alone. Another limitation may be the determination of recurrence, which was largely based on documentation and ECG at follow-up, and which can miss asymptomatic recurrences, although our group also uses web-based routine queries of patients before and after ablation. In future prospective study, we hope to include the entire left atrial appendage (LAA) into the pipeline beyond the LAA ostium only; such differentiation may provide additional insights into the findings reported here.

## Conclusions

In this work we presented a new statistical and machine learning approach to studying AF recurrence via CE-CTA based left atrial atlas differentiation between recurrent and non-recurrent patients. Notable differences in patients who did and did not experience AF recurrence were found in the shape around the left atrial appendage and the pulmonary veins. Additionally, shape features of the LA and areas around the PVs, in conjunction with key clinical factors, were associated with risk of recurrence in AF patients treated with ablation.

## Data Availability

The datasets generated and analyzed during the current study are not publicly available but are available from the corresponding author on reasonable request.

## Abbreviations

CT: Computed tomography
AF: Atrial fibrillation
SOI: Surface of interest
AUC: Area under curve
CE-CTA: Contrast enhanced computed tomography angiography
LA: Left atrium
DiSRn: Differential atlas for identifying sites predictive of recurrence
GLM: General linear model
cSOI: Complement of surface of interest
LAV: Left atrial volume
LVEF: Left ventricular ejection fraction
BMI: Body mass index
PV: Pulmonary veins
LAA: Left atrial appendage
